# The Relationship of Language Barriers to Caregiving Relational Quality among Latino Caregivers

**DOI:** 10.1093/geroni/igaf122.4030

**Published:** 2025-12-31

**Authors:** Michelle Johns, Alan Tiburcio, Gustavo Arruda Franco, Selena Zhong, I’deyah Ricketts, Melissa Howe, Lissette Piedra

**Affiliations:** NORC at the University of Chicago, Chicago, Illinois, United States; NORC at the University of Chicago, Chicago, Illinois, United States; NORC at the University of Chicago, Chicago, Illinois, United States; NORC at the University of Chicago, Chicago, Illinois, United States; NORC at the University of Chicago, Chicago, Illinois, United States; NORC at the University of Chicago, Chicago, Illinois, United States; University of Illinois at Urbana-Champaign, Urbana, Illinois, United States

## Abstract

Sixty-eight million people speak a language other than English at home, with the majority of those speaking Spanish. Language proficiency plays a strong role in shaping individuals’ social networks, and language barriers may affect the quality of an individual’s relationships, such as that between older adults and informal caregivers. Among Latino caregivers of recipients experiencing cognitive decline and coinciding loss of English as a second language, language barriers may intensify deleterious consequences for health and hinder informal caregivers from connecting care recipients to needed healthcare and other formal services. However, to date, few have examined how language differences between caregivers and care recipients shape relationship quality and care dynamics. Using data from a 2025 nationally representative survey of Latino caregivers (n = 625), we assess the prevalence of language barriers as an obstacle to healthcare seeking and the degree to which these barriers affect the relational quality between informal caregivers and older adults. Results indicate high proportions of the Latino caregiving population experiencing language barriers in supporting older adults in accessing healthcare (e.g., 39% reported barriers talking with doctors and other health providers; 31% reported barriers handling health insurance). The presence of these barriers is highly correlated with greater financial, emotional, and physical strain within the caregiving relationship. We discuss results in relation to the growing literature on social diversity in caregiving experiences and interventions (e.g., staff translators on site at medical facilities) that can improve relationship quality between Latino caregivers and health of the older adults in their care.

